# Promising Practices in Caregiving: Learning From Innovative Initiatives Across U.S. States

**DOI:** 10.1093/geroni/igaf122.997

**Published:** 2025-12-31

**Authors:** Rachael Fulp-Cooke, Janice Bell, Quynh Vo, Heather Young

**Affiliations:** Betty Irene Moore School Of Nursing at UC Davis, Sacramento, California, United States; Betty Irene Moore School Of Nursing at UC Davis, Sacramento, California, United States; University of California, Davis, Davis, California, United States; Betty Irene Moore School Of Nursing at UC Davis, Sacramento, California, United States

## Abstract

There is growing recognition of the essential role that family caregivers play in families, communities, and society, as well as the need for comprehensive, tailored support. While California offers some of the most comprehensive publicly funded caregiver services and supports in the country, opportunities remain for the state to better align with priorities outlined in the 2022 National Strategy to Support Family Caregivers. To provide insights to inform California’s approach to caregiving support, we identified innovative and promising caregiving programs and initiatives from other U.S. States with similar population sizes or demographic profiles to California based on the AARP Long-Term Services and Supports Scorecard 2023. As research progressed, additional states with promising practices were identified and included. All initiatives were compiled into an inventory organized by state. Building on a prior analysis of gaps in California’s publicly funded caregiving services and supports, key initiatives addressing the needs of California caregivers were identified. Findings indicate that multiple states have developed and implemented innovative approaches to caregiving support. Notable initiatives from New York, Washington, Texas, and Georgia were recognized as particularly relevant to California’s caregiving landscape. These models offer valuable insights for potential adaptation and enhancement in California and in other settings. Research implications include assessing how these initiatives can be adapted to and integrated into California’s caregiving infrastructure and developing strategies to improve accessibility, sustainability, and effectiveness while strengthening alignment with national priorities.

